# Interannual Variations in Soil Bacterial Community Diversity and Analysis of Influencing Factors During the Restoration Process of *Scirpus Mariqueter* Wetlands

**DOI:** 10.3390/biology14081013

**Published:** 2025-08-07

**Authors:** Yaru Li, Shubo Fang, Qinyi Wang, Pengling Wu, Peimin He, Wei Liu

**Affiliations:** 1 College of Oceanography and Ecological Science, Shanghai Ocean University, Shanghai 201306, China; leah456@163.com (Y.L.); m15152476942@163.com (Q.W.); 18516530305@163.com (P.W.); pmhe@shou.edu.cn (P.H.); 2 Texas Institute for Applied Environmental Research, Tarleton State University, Stephenville, TX 76402, USA; 3 School of Environmental and Chemical Engineering, Shanghai University, Shanghai 200444, China; hsliuwei@shu.edu.cn

**Keywords:** coastal wetland, *Scirpus mariqueter*, high-throughput sequencing, bacterial diversity, soil environmental factor

## Abstract

Due to human activities and the invasion of *Spartina alterniflora*, the population of *Scirpus mariqueter* (*S. mariqueter*) in the Yangtze River Estuary has gradually declined. To address this issue, numerous restoration efforts have been undertaken. To investigate the changes and influencing factors of soil bacterial communities during the restoration of *S. mariqueter* wetlands, we selected *S. mariqueter* populations as the research focus and divided the samples into two years, S1 and S2. High-throughput sequencing technology was employed for observation and analysis. The results revealed that from S1 to S2, soil bacterial diversity in the *S. mariqueter* wetland increased significantly and displayed clear seasonal patterns. The dominant bacterial phyla included Proteobacteria, Bacteroidota, Firmicutes, and Acidobacteriota. Among these, Proteobacteria had the highest relative abundance, while Acidobacteriota showed the most pronounced increase, surpassing Bacteroidota and Firmicutes to become the second most abundant group. Redundancy analysis (RDA) indicated that soil organic matter and electrical conductivity were the key factors influencing the composition and diversity of the soil bacterial community, with Acidobacteriota playing a dominant role during wetland restoration. In conclusion, during the ecological restoration of the *S. mariqueter* wetlands, attention should be given to environmental factors such as soil organic matter and electrical conductivity, while the regulatory role of Acidobacteriota in wetland soils should not be overlooked. This study provides a microscopic perspective on the interactions between microbial diversity and ecosystem functions in coastal wetlands, offering valuable guidance for the ecological restoration of degraded wetlands.

## Simple Summary

The *Scirpus mariqueter* (*S. mariqueter*) wetland has undergone severe degradation due to excessive coastal land reclamation and the invasion of the non-native species *Spartina alterniflora*. In response to this degradation, numerous restoration efforts have been undertaken. During the restoration process, we investigated changes in soil microbial diversity and the key influencing factors. Our findings indicate that soil organic matter and electrical conductivity are the primary drivers shaping the soil bacterial community during the restoration of the *S. mariqueter* wetlands. Additionally, members of the phylum Acidobacteriota were found to dominate in the restored coastal wetland soils. This study offers a theoretical foundation from a microbial perspective for the ecological restoration of degraded wetlands.

## 1. Introduction

Coastal wetlands represent the transitional zones between terrestrial and marine ecosystems. They are essential not only for maintaining ecological security in coastal regions but also for regulating the global climate [1,2]. *Scirpus mariqueter*, a pioneer species in these ecosystems, is primarily distributed across the intertidal zones of the Yangtze River Estuary and Hangzhou Bay. Belonging to the genus *Scirpus* in the family Cyperaceae, it is a perennial herb known for its ability to reduce wave energy, facilitate sediment deposition, stabilize shorelines, and protect embankments [3,4,5]. In addition to its physical functions, *S. mariqueter* holds substantial ecological value. It supports a wide range of wetland wildlife, including waterbirds, fish, benthic invertebrates, and planktonic organisms, thus serving as a critical habitat and food source and contributing significantly to the biodiversity of the Yangtze River Estuary [6]. However, in recent years, the population of *S. mariqueter* has been in decline due to intensive land reclamation and the spread of the invasive species *Spartina alterniflora*. As a result, the restoration of *S. mariqueter* wetlands has emerged as a key focus in the broader effort to rehabilitate coastal wetlands in the Yangtze River Estuary [7,8,9].

Soil microbial communities play a critical role in the decomposition of plant residues and the regulation of biogeochemical processes in wetlands [10,11,12]. Among soil microorganisms in coastal wetlands, bacteria are the most abundant and form the most diverse microbial group, acting as the primary drivers of geochemical cycling in coastal ecosystems [13,14]. Since the launch of a large-scale land reclamation project in 2012 to address land use challenges, the microtopography of the Nanhui Dongtan Wetland in Shanghai has experienced significant alteration. In response, Shanghai Ocean University has undertaken a series of restoration efforts in the *S. mariqueter* wetland at Nanhui Dongtan since 2016. For example, Tao et al. [15] carried out community reconstruction through the transplantation of *S. mariqueter* patches, He et al. [16] conducted a preliminary study on nitrogen allocation, and Zhong et al. [17] and Wang et al. [18] examined the spatiotemporal distribution of large benthic fauna in the *S. mariqueter* community. However, the underlying microbial mechanisms during the restoration process of the Nanhui Dongtan Wetland remain poorly understood.

A growing body of research has revealed strong interactions among wetland vegetation, soil microorganisms, and environmental factors [19,20,21,22,23,24]. Vegetation shifts can influence both the diversity and functional roles of soil microbial communities [25,26], while soil microorganisms are highly sensitive to environmental changes [27,28]. Anthropogenic disturbances, such as land reclamation that depletes *S. mariqueter* populations, can significantly alter the composition of soil bacterial communities. In turn, changes in these microbial communities can impact the structure and function of wetland ecosystems [29]. This raises key scientific questions: How does the soil bacterial community respond during the restoration of the *S. mariqueter* wetlands? What soil factors drive these changes?

Previous studies by Zhao et al. [30], Chi et al. [31], and Guo et al. [32] have shown that soil organic matter, salinity, total phosphorus, and nitrate nitrogen are critical factors influencing the structure and function of soil bacterial communities in coastal wetlands. Based on these findings, we hypothesize that soil organic matter, salinity, total phosphorus, and nitrate nitrogen are the main drivers of changes in soil bacterial communities during the restoration of the *S. mariqueter* wetlands. To test this hypothesis, this study focuses on the *S. mariqueter* wetland in Dongtan, Nanhui. High-throughput sequencing technology was employed to analyze the dynamics of the soil bacterial community and its environmental drivers. The goal is to identify key factors influencing microbial community changes and understand their ecological mechanisms, thereby providing a scientific basis for the effective protection and restoration of degraded coastal wetlands.

## 2. Materials and Methods

### 2.1. Study Area

This study was conducted in the *S. mariqueter* wetland located in Dongtan, Nanhui. The research site lies on the eastern edge of Dongtan, within the Lingang New City district of the Pudong New Area in Shanghai, China (30°51′27″–30°52′10″ N, 121°55′06″–121°56′42″ E). The region experiences a north subtropical monsoon climate characterized by distinct seasons—hot and rainy summers, and mild, humid winters [15]. Annual precipitation exceeds 1000 mm with rainfall concentrated in the summer months, which are also frequently affected by typhoons. The dominant vegetation in the study area consists of *S. mariqueter* communities, with smaller populations of *Phragmites australis* and the invasive *S. alterniflora* growing in proximity [16].

### 2.2. Soil Sample Collection

**Figure 1:**
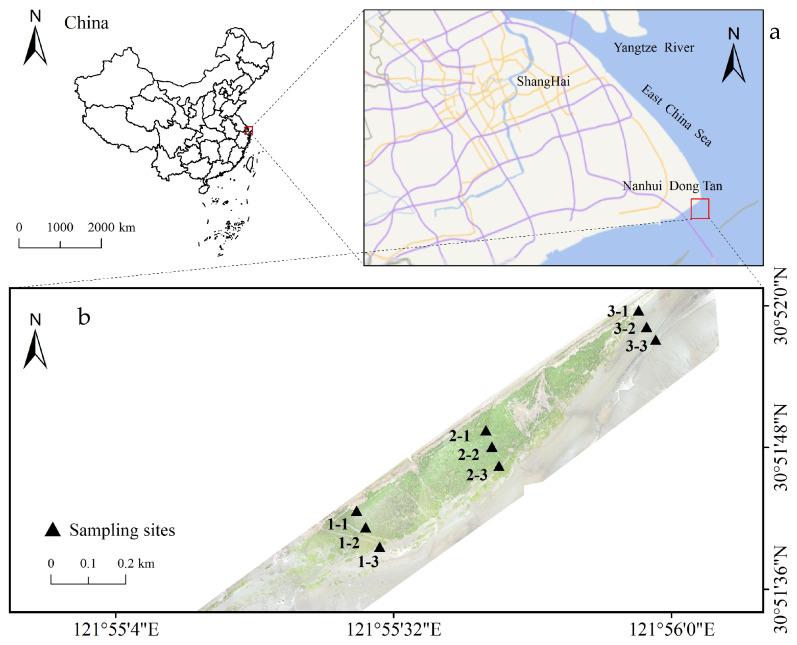
Study area (**a**) and sampling sites (**b**) (the green parts are the plants of the *Scirpus mariqueter* (**b**)); points 1-1, 1-2, and 1-3 are the sparse areas of the *S. mariqueter*; points 2-1, 2-2, and 2-3 are the dense areas of the *S. mariqueter*; points 3-1, 3-2, and 3-3 are the vegetation-free areas of the *S. mariqueter*).

Beginning in May 2019, a field investigation combined with soil sampling and analysis was conducted in the *S. mariqueter* wetland. Based on vegetation density, the study area was categorized into sparse, dense, and vegetation-free zones. Within each zone, fixed sampling points were established at high, medium, and low elevations, resulting in a total of nine sampling points (Figure 1b). Soil samples were collected from each point in May (spring), August (summer), October (autumn), and December (winter) of 2019; October (autumn) and December (winter) of 2020; and May (spring) and August (summer) of 2021.

Sampling followed the five-point method [33], wherein soil blocks were excavated from a 20 cm × 20 cm plot at a depth of 0–20 cm using a shovel. The subsamples were mixed thoroughly and placed into sampling bags. This process was repeated three times per plot. In the laboratory, visible plant roots were removed, and 10 g of soil from each composite sample was placed in a sterile sealed bag for labeling. These samples were stored at −80 °C and sent for centralized microbial analysis [30].

Each month yielded 27 soil samples (9 plots × 3 replicates), resulting in a total of 216 samples across all time points. The remaining soil from each sample was spread on trays to air-dry naturally. Once dried, the soil was passed through a 100-mesh sieve and stored in self-sealing bags for analysis of physical and chemical properties. After laboratory testing, the values for each sampling month were averaged using statistical methods, and the 27 samples from each month were aggregated into one representative sample. Sample identifiers are listed in Table 1.

### 2.3. Determination of Soil Environmental Factors

Soil organic matter (SOM) was measured using the potassium dichromate oxidation-spectrophotometric method (HJ 615-2011). Ammonium nitrogen (NH_4_^+^) and nitrate nitrogen (NO_3_^−^) concentrations were determined using the ultraviolet spectrophotometric method (GB/T 32737-2016). Total phosphorus (TP) was analyzed using the alkaline molybdenum-antimony anti-spectrophotometric method (HJ 632-2011). Soil salinity was represented by soil electrical conductivity (Con), which was measured using a DDSJ-308F conductivity meter (Shanghai Instrument & Electrical Scientific Instrument Co., Ltd., Shanghai, China).

### 2.4. Soil DNA Extraction, PCR Amplification, and Illumina Sequencing

A 500 mg portion of each soil sample was transferred into 2.0 mL tubes containing Lysing Matrix E (a mixture of ceramic and silica particles designed for efficient lysis of soil microorganisms). Homogenization was performed using a FastPrep instrument (MP Biomedicals, Irvine, CA, USA) in the presence of MT Buffer and Sodium Phosphate Buffer. Following lysis, samples were centrifuged into pellet soil particles, cell debris, and the lysing matrix. DNA was then extracted from the supernatant using the Binding Matrix method included in the MP FastDNA™ Spin Kit for Soil (MP Biomedicals, www.mpbio.com), with purification carried out using SPIN filters.

The V3–V4 hypervariable region of the bacterial 16S rRNA gene was amplified using primers 515F and 907R on a GeneAmp^®^ 9700 thermal cycler (Applied Biosystems, Foster city, CA, USA). PCR products were resolved on a 2% agarose gel, and target bands were excised and purified using the AxyPrep DNA Gel Extraction Kit (Axygen Biosciences, Union City, CA, USA). DNA quantification was performed with the QuantiFluor™-ST fluorometer (Promega, Madison, WI, USA) according to the manufacturer’s protocol. Purified amplicons were pooled in equimolar concentrations and sequenced on the Illumina MiSeq platform (Illumina, San Diego, CA, USA) following the standard protocol of Meinuo Biopharmaceuticals Co., Ltd. (Shanghai, China). All sequencing and related laboratory procedures were performed by Meiji Biopharmaceuticals (Shanghai, China).

### 2.5. Statistical Analyses

Operational taxonomic units (OTUs) were clustered at a 97% sequence similarity threshold using the UPARSE software platform (version 7.0.1090; http://drive5.com/uparse/, accessed on 12 June 2023). Taxonomic classification of OTU representative sequences was performed using the RDP Classifier Bayesian algorithm against the SILVA database (Release 138; http://www.arb-silva.de, accessed on 24 June 2023). Community composition for each sample was statistically analyzed at the phylum level.

Alpha diversity indices of the soil bacterial communities—including the ACE, Chao1, Shannon, and Simpson indices—were calculated using Mothur software (version 1.30.2; https://mothur.org/wiki/calculators/, accessed on 5 July 2023). To test for significant differences in alpha diversity and bacterial community composition between the S1 and S2 sampling years, independent-sample *t*-test were conducted using the boot (version 1.3.18) and stats (version 3.3.1) packages in R software (version 3.3.1). Bar charts of soil bacterial community composition were also generated in R to visualize shifts in community structure.

Soil physicochemical parameters were statistically analyzed using Microsoft Excel. Differences in average soil factor values between S1 and S2 were evaluated using *t*-test in SPSS software (version 26.0; IBM Corp., Armonk, NY, USA). Redundancy analysis (RDA) was conducted using Canoco 5.0 (Microcomputer Power, Ithaca, NY, USA) to explore the relationships between soil bacterial community composition, diversity indices, and soil environmental factors. The statistical significance of the RDA was assessed by Monte Carlo permutation tests with 499 permutations (*p* < 0.05).

## 3. Results

### 3.1. Soil Environmental Factors

The results of soil environmental factor measurements in the *S. mariqueter* wetland are presented in Table 2. Overall, the soil organic matter (SOM) content in the study area was relatively low compared with other coastal wetlands. However, a significant increase in SOM was observed from year S1 to S2, rising from 3.94 g/kg to 11.79 g/kg (*p* < 0.001). The soil nitrogen and phosphorus conditions were generally favorable. Notably, nitrate nitrogen (NO_3_^−^) content decreased significantly from 6.95 mg/kg in S1 to 1.01 mg/kg in S2 (*p* < 0.05). Although ammonium nitrogen (NH_4_^+^) and total phosphorus (TP) levels also declined to some extent, these changes were not statistically significant (*p* > 0.05). Soil electrical conductivity (EC) remained within normal ranges, with no significant difference detected between the two years (*p* > 0.05).

### 3.2. Diversity and Composition of Soil Bacterial Communities

#### 3.2.1. Analysis of Soil Bacterial Community Diversity

As shown in Table 3, the ACE, Shannon, and Chao1 indices of soil bacterial communities in the *S. mariqueter* wetland significantly increased from year S1 to S2, while the Simpson index showed a significant decrease. Overall, the Shannon and Chao1 indices were higher in autumn and winter, whereas the Simpson index was lower during these seasons, indicating that soil bacterial diversity exhibits clear seasonal variations in the *S. mariqueter* wetland.

**Figure 2:**
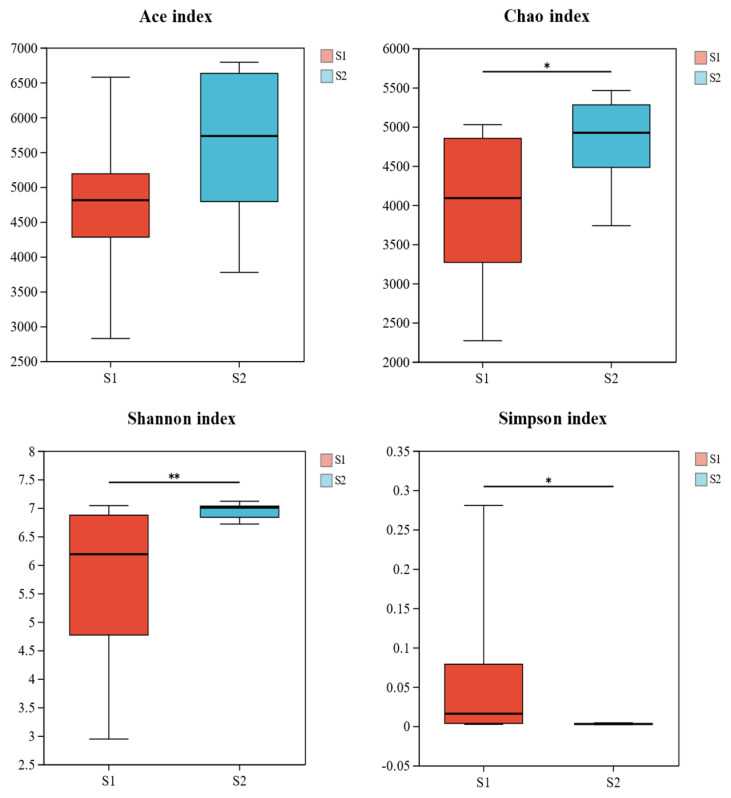
Box plot of soil bacterial α diversity index of *Scirpus mariqueter* wetland. * *p* < 0.05, level of significance difference. ** *p* < 0.01, level of significance difference.

The *t*-test results (Figure 2) revealed significant differences in the Simpson and Chao1 indices between S1 and S2 (*p* < 0.05) and a highly significant difference in the Shannon index (*p* < 0.01). No significant difference was observed for the ACE index (*p* > 0.05). These results suggest that the restoration process of the *S. mariqueter* wetland has led to a significant increase in soil bacterial diversity, with diversity patterns influenced by seasonal changes.

#### 3.2.2. Analysis of Soil Bacterial Community Composition

**Figure 3:**
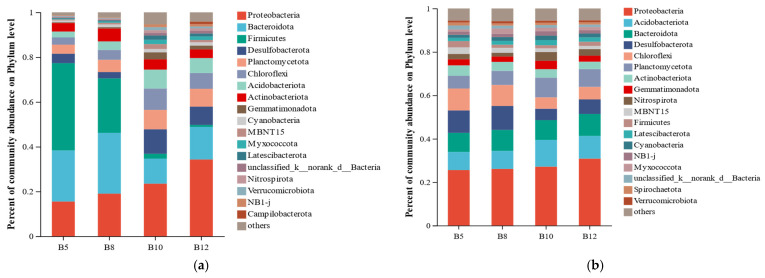
Soil bacterial composition S1 (**a**) and S2 (**b**) of *Scirpus mariqueter* wetland.

The composition of the soil bacterial community at the phylum level is shown in Figure 3. In the S1-year samples (Figure 3a), the dominant phyla were Proteobacteria (23.08%), Bacteroidota (18.91%), Firmicutes (16.58%), Desulfobacterota (6.54%), Planctomycetota (6.54%), Chloroflexi (6.06%), Acidobacteriota (5.40%), and Actinobacteriota (4.47%). Among these, Proteobacteria, Bacteroidota, and Firmicutes were the most abundant, indicating their dominance in the *S. mariqueter* wetland at that time.

In contrast, the S2-year samples (Figure 3b) were dominated by Proteobacteria (27.41%), Acidobacteriota (9.86%), Bacteroidota (9.42%), Desulfobacterota (8.33%), Chloroflexi (7.70%), Planctomycetota (7.41%), Actinobacteriota (4.06%), and Gemmatimonadota (2.98%). The most abundant groups in S2 were Proteobacteria, Acidobacteriota, and Bacteroidota.

Compared with the S1 samples, the composition of the dominant bacterial phyla shifted noticeably in S2, particularly with a marked increase in the relative abundance of Acidobacteriota and a decrease in Firmicutes, suggesting changes in the soil microbial community structure during the restoration process (Figure 3b).

**Figure 4:**
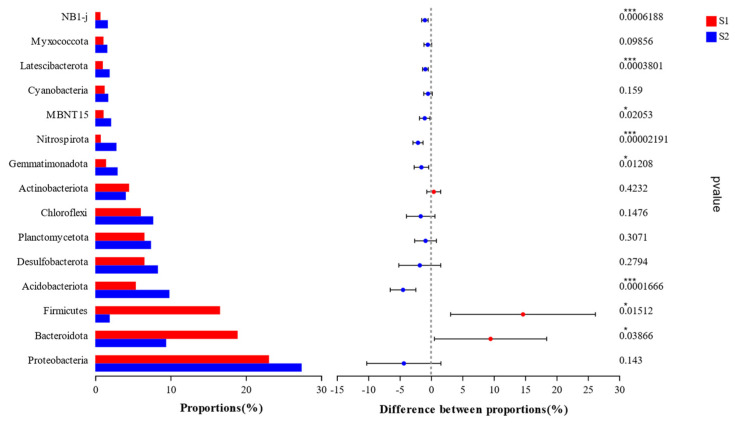
Interannual bacterial abundance difference between S1 and S2 (Student’s *t*-test bar plot on phylum level). * *p* < 0.05, level of significance difference. *** *p* < 0.001, level of significance difference.

As shown in Figure 4, the relative abundance of Acidobacteriota significantly increased in the S2-year samples compared with S1 (*p* < 0.001), while the relative abundance of Firmicutes significantly decreased (*p* < 0.05). This shift suggests that Acidobacteriota has replaced Firmicutes as one of the dominant soil bacterial groups in the wetland. Additionally, the relative abundance of Bacteroidota, another dominant phylum, also declined significantly in S2 (*p* < 0.05). In contrast, no significant differences were observed in the relative abundances of other major bacterial phyla—including Proteobacteria, Desulfobacterota, Planctomycetota, Chloroflexi, and Actinobacteriota—between the two years (*p* > 0.05).

### 3.3. Correlation Between Soil Environmental Factors and Soil Bacterial Communities

**Figure 5:**
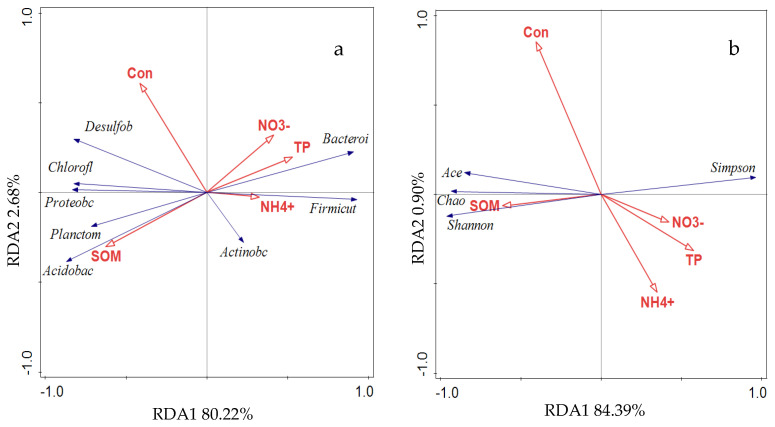
Soil microbial community composition (**a**), diversity index (**b**), and soil environmental factor redundancy analysis. (The explanatory variables are shown by different arrows: blue solid arrows show soil bacterial community composition—Proteobacteria (Proteobc), Bacteroidota (Bacteroi), Firmicutes (Firmicut), Acidobacteriota (Acidobac), Desulfobacterota (Desulfob), Planctomycetota (Planctom), Chloroflexi (Chlorofl), Actinobacteriota (Actinobc)—and soil bacterial diversity index—Ace, Chao, Shannon and Simpson; red arrows show environmental variables—soil organic matter (SOM), electrical conductivity (Con), soil nitrate nitrogen (NO_3_^−^), soil ammonia nitrogen (NH_4_^+^), and soil total phosphorus (TP)).

Redundancy analysis (RDA) was conducted to explore the relationships between environmental factors and soil bacterial communities (Figure 5). The RDA of environmental variables and bacterial community composition at the phylum level revealed that the first two axes accounted for 82.9% of the total variance (RDA1 = 80.22%, RDA2 = 2.68%). Soil organic matter was identified as the primary driver of variation in bacterial community structure, with nitrate nitrogen (NO_3_^−^) also contributing significantly to community composition (Figure 5a). The dominant phylum Acidobacteriota showed a strong positive correlation with soil organic matter and a negative correlation with Firmicutes and Bacteroidota. Conversely, Firmicutes and Bacteroidota were positively correlated with NO_3_^−^ levels.

RDA of environmental variables and soil bacterial alpha diversity indices indicated that the first two axes explained 85.29% of the total variance (RDA1 = 84.39%, RDA2 = 0.90%) (Figure 5b). Soil electrical conductivity and soil organic matter were identified as the main factors influencing the diversity and distribution of soil bacterial communities at the phylum level. Among the diversity indices, the ACE, Shannon, and Chao1 indices were strongly positively correlated with soil organic matter. In contrast, the Simpson index was positively correlated with NO_3_^−^ and strongly negatively correlated with soil electrical conductivity.

## 4. Discussion

### 4.1. Characteristics of Changes in Soil Influencing Factors

The *S. mariqueter* wetland is located in the Dongtan area of Nanhui District, Shanghai. As a reclaimed coastal area, it functions as a seaport and is not used for agricultural purposes. There are few industrial facilities in the vicinity; however, the area is surrounded by residential communities. Some domestic sewage discharged from nearby households flows into the wetland, and recreational activities by tourists contribute to environmental stress through littering. Moreover, large-scale land reclamation projects initiated by the Shanghai Municipal Government have caused extensive damage to wetland vegetation and severely degraded the soil structure [34].

As shown in Table 2, during the S1 sampling year, the soil organic matter (SOM) content was relatively low, while nitrogen and phosphorus levels were relatively high. This can be attributed to persistent anthropogenic disturbances prior to 2020. However, during the COVID-19 lockdown in 2020, reclamation activities were suspended, and residents were confined to their homes, resulting in a significant reduction in human disturbance. Under these conditions, *S. mariqueter* vegetation regenerated and proliferated. Previous studies have demonstrated that *S. mariqueter* can substantially increase SOM levels while effectively reducing soil nitrogen and phosphorus concentrations [15]. Accordingly, by the S2 sampling year, SOM content had increased significantly, while nitrate nitrogen levels had decreased.

In coastal wetland ecosystems, soil electrical conductivity is largely influenced by hydrological dynamics and salinity gradients [35]. The slight increase in electrical conductivity observed in the S2 samples may be attributed to seawater intrusion.

### 4.2. The Diversity and Composition Structure Changes in Soil Bacterial Communities

Species diversity is a central theme in ecology, and diversity indices are commonly used to characterize the complexity and richness of biological communities [36,37,38]. As shown in the soil bacterial diversity indices and supported by *t*-test results, the *S. mariqueter* wetland exhibited a significant increase in both bacterial diversity and richness following ecological restoration. Additionally, bacterial diversity was higher in autumn and winter compared with spring and summer, a pattern consistent with previous studies [30].

The accelerated restoration of the wetland is likely attributable to the reduction in human disturbance during the COVID-19 lockdown in 2020. With the suspension of reclamation activities and fewer visitors, *S. mariqueter* vegetation recovered rapidly. This species possesses a well-developed root system, and after tidal retreat, large numbers of plankton and invertebrates remain trapped in the root zone. In the absence of human interference, these organisms are able to forage, reproduce, and metabolize within the soil matrix surrounding the plant roots [39]. Such activity promotes microbial colonization in the rhizosphere, enhancing soil bacterial richness and community diversity.

Although microbial abundance in coastal wetlands typically peaks in summer and autumn due to favorable temperature and moisture conditions [40], this study found higher bacterial diversity in autumn and winter. This discrepancy may be explained by plant–microbe interactions. In spring and summer, vigorous plant growth leads to increased uptake of soil nutrients and carbon, potentially resulting in competition between plants and soil microbes [41]. The reduced availability of organic substrates to microbes may limit microbial proliferation. In contrast, during winter, plant senescence and decomposition increase the availability of organic matter, promoting microbial growth and leading to greater bacterial richness and diversity. Furthermore, frequent summer typhoons in the wetland may contribute to the observed decline in bacterial diversity during that season by damaging vegetation and reducing populations of benthic fauna [42].

Soil bacterial communities play an essential role in sustaining the health and ecological functioning of wetland ecosystems. Previous studies have shown that the bacterial communities in coastal wetland soil are predominantly composed of Proteobacteria and Bacteroidota [43]. As illustrated in Figure 3, the dominant phyla identified in the *S. mariqueter* wetland were Proteobacteria, Bacteroidota, Firmicutes, and Acidobacteriota, consistent with earlier findings on coastal wetland bacterial communities. This study observed a notable shift in dominant bacterial phyla between the S1 and S2 sampling years. Specifically, the relative abundance of Firmicutes significantly decreased, while that of Acidobacteriota significantly increased, making Acidobacteriota the second most abundant phylum in the community by S2. Numerous studies have reported that Acidobacteriota plays a key role in biogeochemical cycling and the development of soil ecosystems. Its abundance is often strongly positively correlated with soil organic matter content [44,45]. These findings suggest that the observed increase in Acidobacteriota may be driven by the significant rise in soil organic matter recorded during the restoration process, highlighting its ecological relevance in soil nutrient cycling and microbial succession within the *S. mariqueter* wetland.

### 4.3. Correlation Analysis of Soil Bacterial Communities and Soil Environmental Factors

Salinity and organic matter are two key environmental factors that strongly influence the structure and function of soil microbial communities [31,46]. In this study, soil organic matter (SOM) was found to be highly positively correlated with both the Shannon and Chao1 indices, indicating that increases in SOM significantly enhance soil bacterial diversity in the *S. mariqueter* wetland. Elevated SOM levels provide essential nutrients for microbial survival, stimulate growth and reproduction, and improve metabolic activity [47], thereby supporting greater microbial richness and diversity.

RDA further revealed strong positive correlations between SOM and dominant bacterial phyla such as Acidobacteriota, Proteobacteria, and Planctomycetota. Previous studies have demonstrated the involvement of Acidobacteriota in the carbon cycle, particularly in the decomposition of litter and detritus in wetland soil [48]. Thus, the increase in SOM likely promotes the enrichment of Acidobacteriota, confirming its role as a key functional group during ecological restoration. Collectively, these findings support the conclusion that soil organic matter is a major driver of both community composition and microbial diversity in the *S. mariqueter* wetland.

Soil electrical conductivity (EC), which serves as an indirect indicator of salinity, was also identified as an important environmental factor. Prior research has shown that salinity significantly shapes bacterial abundance and distribution patterns [49,50], consistent with observations at the phylum level in this study. RDA results indicated a strong positive correlation between EC and Desulfobacterota, Proteobacteria, and Chloroflexi, while correlations with Actinobacteriota and Acidobacteriota were relatively weak. In contrast, Firmicutes and Bacteroidota exhibited strong negative correlations with EC, suggesting that their decline may be linked to increased salinity. These patterns highlight the differential response of bacterial phyla to salinity stress and suggest that EC influences the horizontal distribution and structural reorganization of soil bacterial communities.

Moreover, increases in EC were positively correlated with the ACE and Chao1 diversity indices, and negatively correlated with the Simpson index. This suggests that salinity may favor the proliferation of halophilic or salt-tolerant taxa, thereby increasing community diversity. For example, Gemmatimonadota is known to prefer saline environments, and its abundance was positively associated with higher EC levels in this study [32,51].

In summary, both soil organic matter and electrical conductivity emerged as key factors shaping the composition and diversity of soil bacterial communities in the *S. mariqueter* wetland. However, our understanding of the competitive dynamics among dominant phyla remains limited. Future studies should incorporate metagenomic approaches to analyze functional gene expression and elucidate inter-phylum competition and cooperation mechanisms. Such insights would enhance our understanding of microbial community evolution during wetland restoration and support the development of micro-scale restoration strategies, including the targeted use of microbial inoculants to rehabilitate degraded coastal wetlands.

## 5. Conclusions

This study reveals the dynamics of soil bacterial diversity and community composition, as well as the key soil factors influencing these changes during the ecological restoration of the *S. mariqueter* wetland. From S1 to S2, soil bacterial diversity increased significantly and exhibited clear seasonal variation. The composition of dominant bacterial phyla also shifted, with Acidobacteriota showing the most notable increase and emerging as the second most abundant phylum in the wetland soil community. Redundancy analysis (RDA) further identified soil organic matter and electrical conductivity as the primary environmental drivers of these microbial changes during the restoration process.

These findings provide valuable insights into the patterns and drivers of bacterial community structure in coastal wetlands and offer a microscopic perspective on the ecological processes underlying wetland restoration. Understanding these microbial dynamics is essential for supporting the long-term ecological stability and functional recovery of degraded coastal wetland ecosystems.

## Figures and Tables

**Table 1:** Sample number table.

Anniversary Number	Sampling Time	Sample Number
S1	May 2019	19-5
	August 2019	19-8
	October 2019	19-10
	December 2019	19-12
S2	October 2020	20-10
	December 2020	20-12
	May 2021	21-5
	August 2021	21-8

**Table 2:** Determination of soil physical and chemical properties of the *Scirpus mariqueter* wetland.

Year	Samples	SOM/ (g/kg)	NH_4_^+^/(mg/kg)	NO_3_^−^/(mg/kg)	TP/(g/kg)	Con/(µs/cm)
S1	19-5	3.99	16.13	8.31	0.61	900.40
	19-8	2.45	14.24	4.95	0.52	602.20
	19-10	1.40	22.34	13.04	0.64	538.80
	19-12	7.93	6.91	1.49	0.38	1948.00
	Mean	3.94	14.91	6.95	0.54	997.35
S2	20-10	12.37	9.95	0.66	0.36	1201.33
	20-12	11.00	12.54	1.71	0.47	1051.67
	21-5	10.94	13.39	0.70	0.47	758.11
	21-8	12.84	9.01	0.97	0.32	1026.39
	Mean	11.79	11.22	1.01	0.41	1009.37
	*p*	0.002 **	0.313	0.031 *	0.107	0.973

Note: * *p* < 0.05, level of significance difference. ** *p* < 0.01, level of significance difference.

**Table 3:** Index of soil bacterial diversity of *Scirpus mariqueter* wetland.

Year	Samples	Ace	Shannon	Simpson	Chao
S1	19-5	3877.225	3.983	0.173	2965.878
	19-8	4724.559	4.711	0.113	3515.368
	19-10	5932.280	6.996	0.002	4922.999
	19-12	6385.339	6.833	0.004	5190.885
	Mean	5229.851	5.631	0.073	4148.783
S2	20-10	5091.273	6.956	0.003	4562.520
	20-12	5908.541	6.963	0.003	4902.821
	21-5	6264.320	6.965	0.003	5109.963
	21-8	6889.622	7.060	0.002	5518.087
	Mean	6038.439	6.986	0.003	5023.348

## Data Availability

Data are available from the corresponding author upon reasonable request.
